# Instruments for reproducible setting of defects in cartilage and harvesting of osteochondral plugs for standardisation of preclinical tests for articular cartilage regeneration

**DOI:** 10.1186/s13018-015-0257-x

**Published:** 2015-07-28

**Authors:** Markus L. Schwarz, Barbara Schneider-Wald, Joachim Brade, Dieter Schleich, Andy Schütte, Gregor Reisig

**Affiliations:** Section for Experimental Orthopaedics and Trauma Surgery, Orthopaedic and Trauma Surgery Centre (OUZ), University Medical Centre Mannheim, Theodor-Kutzer-Ufer 1-3, 68167 Mannheim, Germany; Department of Medical Statistics, Biomathematics and Information Processing, Medical Faculty Mannheim, University of Heidelberg, Mannheim, Germany

## Abstract

**Background:**

Standardisation is required in research, so are approval procedures for advanced therapy medical products and other procedures for articular cartilage therapies. The process of creating samples needs to be reproducible.

The aim of this study was to design, create and validate instruments (1) to create reproducible and accurate defects and (2) to isolate samples in the shape of osteochondral cylinders in a quick, reliable and sterile manner.

**Methods:**

Adjustable instruments were created: a crown mill with a resolution of 0.05 mm and a front mill to create defects in articular cartilage and subchondral bone. The instruments were tested on knee joints of pigs from the slaughterhouse; 48 defects were created and evaluated. A punching machine was designed to harvest osteochondral plugs. These were validated in an in vivo animal study.

**Results:**

The instruments respect the desired depth of 0.5 and 1.5 mm when creating the defects, depending on whether the person using the instrument is highly experienced (0.451 mm; confidence interval (CI): 0.390 mm; 0.512 mm and 1.403 mm; CI: 1.305 mm; 1.502 mm) or less so (0.369 mm; CI: 0.297 mm; 0.440 mm and 1.241 mm; CI: 1.141 mm; 1.341 mm). Eighty samples were taken from knee joints of Göttingen Minipigs with this punching technique. The time needed for the harvesting of the samples was 7.52 min (±2.18 min), the parallelism of the sides of the cylinders deviated by −0.63° (CI: −1.33°; 0.08°) and the surface of the cartilage deviated from the perpendicularity by 4.86° (CI: 4.154°; 5.573°). In all assessed cases, a sterile procedure was observed.

**Conclusions:**

Instruments and procedures for standardised creation and validation of defects in articular cartilage and subchondral bone were designed. Harvesting of samples in the shape of osteochondral cylinders can now be performed in a quick, reliable and sterile manner. The presented instruments and procedures can serve as helpful steps towards standardised operating procedures in the field of regenerative therapies of articular cartilage in research and for regulatory requirements.

## Background

Guidelines for the determination of therapeutic outcomes require reproducible and validated protocols to be observed. Standards would also be helpful to satisfy regulatory requirements for regeneration therapy of articular cartilage tissue in preclinical tests in vivo and in vitro [1, 2].

There are some specific challenges in preparing and isolating samples before and after the healing time regarding different types of analyses and the stated scientific hypotheses. Biomechanical tests in particular require specimens with a clear geometry. This is necessary in order to keep the specimen in place and allow free access for biomechanical testing devices such as indentation rods [3–6]. The same is true for specimens used in tribological test procedures [7–12]. Thus, the exact definition and isolation from surrounding tissue of the specimen is vital for reproducible results.

For the synthesis of regenerated articular cartilage tissue, a standardisation of the procedures is required. Most of the studies use defect models analysing regenerative concepts [13–16], but the procedures are rarely harmonised at the level of preparation. The operative procedures follow the clinical requirement that smooth defect edges, consisting of stable cartilage tissue, lie perpendicular to the bottom of the defect [17, 18]. Different procedures are described and validated for the debridement of fibrous and degenerative tissue prior to the treatment with autologous chondrocyte implantation (ACI) or Matrix-based ACI [19]. Referring to published results and our own tests, the question arises if the frequently used curette can deliver the requested outcomes regarding the quality of the created articular defects in terms of the geometry and resection of the tissue [19, 20].

The regeneration of defects in the articular cartilage is described and investigated among others by different depths according to the hypothesis of the particular study design. One can roughly distinguish between partial and full thickness defects [21] which can be extended into the bone in order to open the subchondral lamella to enable stem cells to migrate into the defect [22] or in order to prepare osteochondral autologous transplantation [23].

However, the depth of the defects should be stable in dimension, homogenous in shape and conform to the same principle. The sides should be straight and the bottom of the defect should be perpendicular to the sides.

Therefore, a cutting device is required which produces smooth surfaces on the cartilage and the bone. It must be capable of working in a quick, reproducible manner and irrespective of the user’s expertise in order to guarantee standardisation in an in vivo model to produce advanced therapy medicinal product (ATMP). It also needs to be suited for scientific assessment and has to meet regulatory guidelines.

In the field of research of tissue engineering of articular cartilage, osteochondral blocks or plugs are often used with the subchondral bone as a fixation and the cartilage layer on top as protruding tissue to be examined. Thus, the isolation of samples can become a challenge when new tissue has to be isolated for testing. The time for preparation should be as short as possible to avoid changes arising after opening the joint.

Samples for biomechanical and other tests have to be of a specific size, depending on the test procedure. Several biomechanical tests require samples of a size varying between 4 mm [24] and 6 mm [4] in diameter. Therefore, large animal models have an advantage over small ones in this context also for measuring the outcome in osteoarthritis (OA) research [25]. Sheep, goats, dogs, horses, pigs and also rabbits are mainly used in studies dealing with regenerative therapies for articular cartilage [26, 13, 27–30].

The aim of the study was to establish a reproducible design to create and isolate regenerative articular cartilage samples in large animals applicable in and ex vivo and in vitro.

In particular, instruments had to be designed and constructed allowing (1) the creation of defects and (2) the isolation of the area of the regenerated tissue after healing. The operating sequence can also be applied to other species, either in vitro, in vivo and ex vivo.

## Material and methods

### Tool for creating defects

We constructed and built tools for the creation of reproducible chondral or osteochondral defects in vivo and in vitro in joints of animals.

**Fig. 1:**
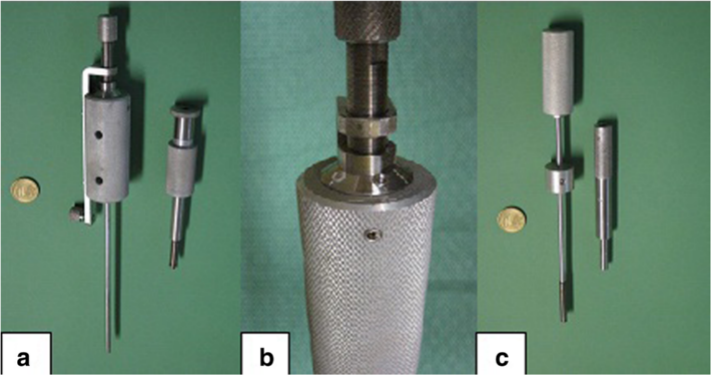
The instruments for creating defects in articular cartilage. **a** The disassembled crown mill—*left* the inner rod, *right* the cutting device—is equipped with a hand screw on top (**b**) for adjusting the desired depth of the circular groove. **b** The depth of the defect can be adjusted with a resolution of 50 μm using the scale with 10 steps. **c** The front mill consists of two parts, the cutting device (*left*) and the sleeve with an outer diameter of 6 mm that fits exactly into the circular groove prepared by the crown mill. Both instruments need to be turned clockwise

**Fig. 2:**
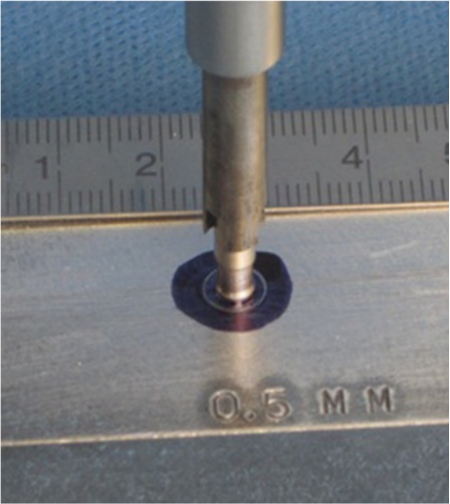
Calibration of the crown mill. The crown mill needs to be calibrated before adjusting the desired depth. In the above picture, the pegs of the centralizer of the crown mill are placed in a 0.5-mm cavity of the calibration tool providing various cavities with different depths. The starting point is reached by gently turning the cutting blades until the black mark is touched. The depth of calibration represents the expected depth of penetration of the pegs into the cartilage tissue under pressure. This is necessary as the penetration depth has to be considered when the final depth of the defect is adjusted

**Fig. 3:**
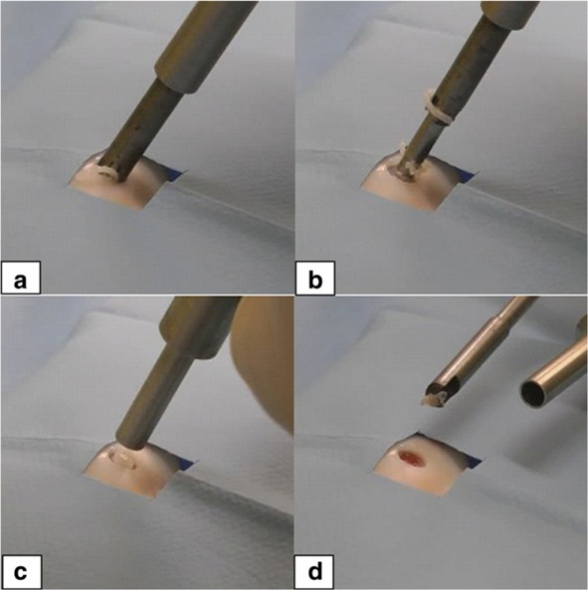
Creating a defect in cartilage tissue. **a** The crown mill is centred with the help of the pegs and the cutting process can be started. **b** When the adjusted depth is reached, a further feed is blocked even if the instrument is turned. Notice the proper chip formation, due to the customised profile of the blades. **c** The outer sleeve of the front mill is placed in the circular groove made by the crown mill. **d** The cartilage plug in the middle is cut by the front mill, thus creating a smooth surface. The harvested cartilage tissue can be used for the isolation of chondrocytes for ACT or MACT

The instrument for creating the defects in articular cartilage consists of two different tools (Fig. 1). The so-called “crown mill” is used at first and creates a circular groove with an outer diameter of 6 mm and an inner diameter of 4 mm resulting from the thickness of the circular cutting blade of 1 mm (Fig.1a, b). Findings show that a defect size of 6 mm in diameter is critical as spontaneous healing is not likely to occur in large animal models such as pigs, goats and dogs [13, 14]. We decided on the trochlea of the Göttingen Minipig, because the width of one facet tolerates a 6 mm defect and its length at least two defects. The so-called “front mill” removes the inner sector of the defect and is used after the crown mill (Fig. 1c). The crown mill can be adjusted according to the desired depth of the defect in 0.05-mm steps resulting from a thread with a lead of 0.5 mm for each turn (Fig. 1a). The inner rod of the crown mill has four pegs at the top with 0.6 mm in height, which can be pressed into the cartilage tissue, thus preventing a displacement. With a calibration tool different depth of penetration of the pegs can be adjusted before use (Fig. 2). Afterwards, the tool is positioned at the desired surface point of the joint, and the user turns the outer part of the tool, which is centred by the inner rod, clockwise into the tissue (Fig. 3). Thus, a circular groove is created and the position and depth of the defect are defined.

The outer sleeve of the front mill is placed in the groove and the inner section is turned clockwise, until the inbuilt stop respects the depth defined by the crown mill (Fig. 3c, d). Residual tissue at the bottom of the defect can be removed with the front mill or a curette. A curette (Uteruskürette 4.5 ml, Aesculap, Tuttlingen, Germany) was sharpened by the toolmaker for this purpose.

### Validation of the defect tool

The hypothesis for the validation of the newly created instrument was twofold:

Firstly, we wanted to investigate if the tool could deliver the expected depth of a defect in articular cartilage or bone after the desired dimension was preset at the instrument. Secondly, differences between an experienced and a less experienced practised operator were to be addressed.

To validate the tool for creating defects with defined depths, we used trochleae of the knee joints of pigs (age approximately 5 months) from the local slaughterhouse. Defects with 0.5 mm (*n* = 12) and 1.5 mm (*n* = 12) in depth, respectively, were placed at random in each trochlea.

Two operators created a total of 48 defects in 12 trochleae, using the same tool.

One operator was a highly experienced operator (toolmaker DS); the other one was a scientist (biologist GR) with an assumed lower skill level in using tools.

**Fig. 4:**
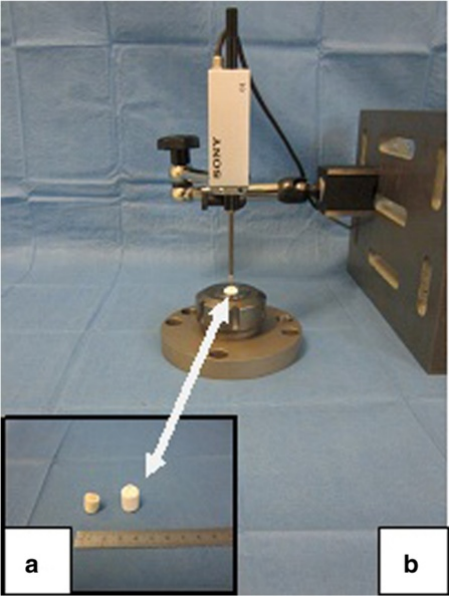
Measurement of the defects. **a** The measurement of the defects was performed with casts made out of dental cement (detail, *right*) which were taken from the moulds made of alginate, (detail, *left*) thus representing the original shape of the defects. **b** The solid cast (*arrow*) is fixated in a tap holder. Thus, it was possible to take the measurements of the defects indirectly but exactly with a contacting digital gauge

The defects were cast with alginate (Alginat Protesil ®Vannini Dental Industry, Grassini, Italy). As the stability of alginate is limited, a second cast with dental cement (Super Rock Dental Stone, Noritake Co, Japan) was performed at the top of the alginate body. Thus, the defects became solid enough to be able to perform a measuring procedure to determine their depths, thus avoiding measuring faults through contact with a digital gauge (DT32P, Sony Precision Technology Inc., Tokyo, Japan). The casts were mounted in a tap holder which was adjusted to the vertically positioned digital gauge for measuring (Fig. 4).

The measurement algorithm was based on the idea that the first groove made by the crown mill represents the point of reference. Thus, the area in the middle of the defect that was created by the front mill and the untouched cartilage surface can be calculated as the difference in height according to the point of reference. The defects were measured in four positions (12, 3, 6 and 9 o’clock) at the three levels (circular groove, untouched articular surface and middle of the defect) three times. Taking the means of the triplicates, the average depth of one defect was calculated with regard to the depth of the groove created by the crown mill and the area created by the front mill. The measurements of the twelve defects with the same defined depth provided the average depth and the corresponding statistical deviation.

### Statistics

The depths were calculated by Excel 2010 (Microsoft® Corporation, Redmond, USA) in terms of differences, and the values of the results are shown graphically by box blots created by the Origin 8.6G software (OriginLab Corporation, Northampton, USA). Differences in the results of both operators were tested by the *t* test procedure in the SAS 9.3 program (SAS® Institute, Cary, USA). The level of significance was *α* = 0.05.

### Harvesting the samples after healing

There are some issues to be considered and overcome when osteochondral samples have to be taken out of the joint, in particular from the bone after a longer healing period.

The articular cartilage layer is supported by the subchondral bone lamella which can develop into a rigid structure [31–33] during OA. This structure can occur in an in vivo experiment after a healing time of up to 1 year or even less. In preliminary tests, we experienced that the preparation with hammer and chisel or punches could result in specimen with uneven geometry regarding the sides and the shape of the desired cylinders (plugs). We noticed that the specimen cannot be stabilised enough and the tool (punch) cannot be kept in position relative to the specimen, as the specimen have to be kept moist to prevent exsiccation. The use of a saw for the separation of specimen causes damage, and the sawdust created by the sawing process can pollute the adjacent surface. Furthermore, the width of the saw has to be taken into account as it needs a layer of tissue between two desired samples, or valuable tissue may be lost in the process. The more defects are created close to one another in one anatomical structure like the facets of the trochlea, the more the distance between them and the surrounding tissue becomes critical. For biomechanical tests, osteochondral plugs are required with isolated regenerated cartilage tissue on the top, to enable e.g. unconfined compression [6] or tribological tests [11]. Thus, the osteochondral plug had to be taken out of the middle of the defect area and frayed edges had to be avoided. A cylindrical shape of the sample guarantees a correct and reproducible way of fixating the plug in fixing devices used for mechanical tests. The plane with the layer (cartilage or cartilage like tissue) on top should be perpendicular to the middle axis of the cylindrical plug. In particular, regarding tribological tests, the surface of the regenerated or cartilage tissue has to be protected against touching or any other outside influence. This has to be taken into account when handling (e. g. while harvesting) the samples. Several types of analyses make the samples sensitive for tissue defects like drying or degradation of proteins. The frictional properties change as well [34]. An important requirement in this context seems to be a quick and safe preparation technique. This is even more important when the analysis requires sterile samples for e.g. cell culture.

The isolation of the samples has to be easy and reliable despite all critical situations described above.

The presented construction for harvesting samples of articular cartilage after healing in vivo is based on a solid frame. A bench vise is mounted which is movable below and allows the adjustment of the fixed sample under the vertically mounted punch in a correct, perpendicular position in relation to the articular surface.

**Fig. 5:**
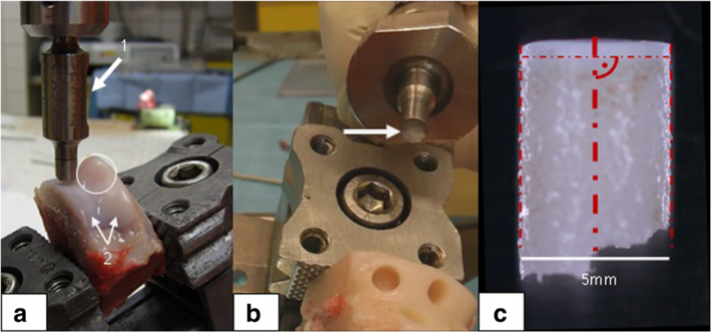
Harvesting of osteochondral plugs. **a** The punch (arrow # 1) is adjusted to take an osteochondral plug from the trochlea of a right knee of a Göttingen Minipig (GM). Four defects on the facets of the trochlea were created previously by the instruments described in Figs. 1 and 8b. The isolated trochlea is fixated between the jaws of a bench vise, and the punch is adjusted over the defect area which is located at the medial facet before punching. The circle indicates the defect area located at the lateral facet after a healing period (regenerated tissue) of 48 weeks^1^. The two distally located defects are not visible. In the proximal parts of the trochlea (arrows # 2), untreated cartilage can serve as control tissue. **b** If the plug stays fixed at the bony bottom, it can be broken out with a hand-held tool, which can be disassembled afterwards to extract the sample (the *arrow* indicates the bony end of the plug with 5 mm in diameter). **c** Osteochondral cylindrical plug from an untreated knee joint (GM) after harvesting with a 5-mm diameter punch serving as control specimen. Guidelines (*red lines*) are drawn in for the analysis of the parallelism of the sides and the perpendicularity of the cartilage surface (*top*) to the perpendicular bisectors of the sides.

In addition, the concentric position of the punch has to be adjusted with reference to the outer rim of the circular defect where the new tissue is expected, and only the new tissue has to be examined. In the presented equipment, the osteochondral plugs which have to be harvested need a diameter of 5 mm, whereas the defect is created with a diameter of 6 mm. The punch is driven into the tissue (cartilage and bone) by hand supported by a lever mechanism (Fig. 5a). We moistened the punch and the specimen with phosphate buffered saline (PBS) in order to reduce friction and avoid the exsiccation of the tissue. If sterile specimens are needed, sterile PBS has to be added. When the desired depth (e. g. approximately 7 mm) has been reached, the punch is retracted to the starting position and the osteochondral plug remains connected with the bone at the bottom. A sleeve with handle is mounted and the osteochondral plug can be taken out by moving it back and forth (Fig 5b). The sleeve can then be disconnected from the hand piece and placed in a special rack in order to extrude the osteochondral plug and place it into a final fixing device (not shown). The plug with the protruding cartilage can then be further manipulated, either in biomechanical tests or by cutting off the cartilage and harvesting the tissue alone if needed.

In some cases, the osteochondral plug remains in the punch when retracting it. The punch (Fig. 5a) is equipped with a kind of window through which the plug can be taken out with tweezers. A rod is inserted at the bony end of the plug and driven out by gentle blows with a hammer; thus, the surface of the cartilage remains untouched.

### Validation of the harvesting tool

We applied the harvesting tools in a study using a large animal model with artificial articular defects in both facets of the trochlea at both sides according to Gotterbarm et al. [14]. The study was approved by the ethical committee number 35-9185.81/G-6/11 (Regierungspräsidium Karlsruhe, Germany).

We determined (1) the time needed to create an osteochondral plug, (2) the parallelism of the sides of the plug, (3) the deviation of its surface from its symmetric axis that should be 0° regarding the addressed 90°, and (4) the sterility of the procedure if sterile samples are needed.

We started timing when the adjustment of the defect to the punch above began after the separated trochlea was fixated in the bench clamp and stopped the time when the osteochondral plug was positioned under the camera taking pictures (body: Canon EOS 7D; lens: Canon EF 100 mm/2.8 L; Canon, Tokyo, Japan) for analysing the shape and geometry of the plug. The camera was mounted on a frame (camera stand RSX, arm RTX and lightning device RB5000; Kaiser Fototechnik GmbH und Co.KG, Buchen, Germany) parallel to the base where the plug was placed on its side. Thus, the optical-parallaxes were avoided, and a contactless measurement became possible.

The geometry of the osteochondral plugs was analysed through the pictures taken after harvesting. For this purpose, pictures were taken from four different angles, and a particular view of the side of the plug that seemed the least straight was taken for evaluation. The geometry of the plug was analysed with the OpenOffice 3.4 Draw program (Apache Software Foundation, Forest Hill MD, USA).

Both sides of the plug form a positive angle towards the cartilage at the top if the tangents at the sides converge, a negative angle vice versa.

Eighty plugs, harvested from both trochleae of 10 Göttingen Minipigs, were analysed. Sterility was checked by swabs (Copan Diagnostics Inc. Murrieta CA, US or Nerbeplus, Winsen/Luhe, Germany) at the surfaces of the remaining trochlea and analysed. The swabs were incubated according to the microbiological and infectiological quality standards (Mikrobiologisch-infektiologische Qualitätsstandarts (MiQ)) of the German Society for Hygiene and Microbiology (Deutsche Gesellschaft für Hygiene und Mikrobiologie (DGHM)) between 2 and 10 days both in an anaerobic and aerobic environment [35]. Positive results were not detected.

### Statistics

The time needed for harvesting the plugs was calculated in Excel for the mean and standard deviation. We calculated the means and 95 % confidence intervals (CI) by SAS 9.3 for the parallelism of the sides of the plug and the deviation of the edge of the surface of the plugs from their centreline.

## Results

### Validation of the defect tool

**Fig. 6:**
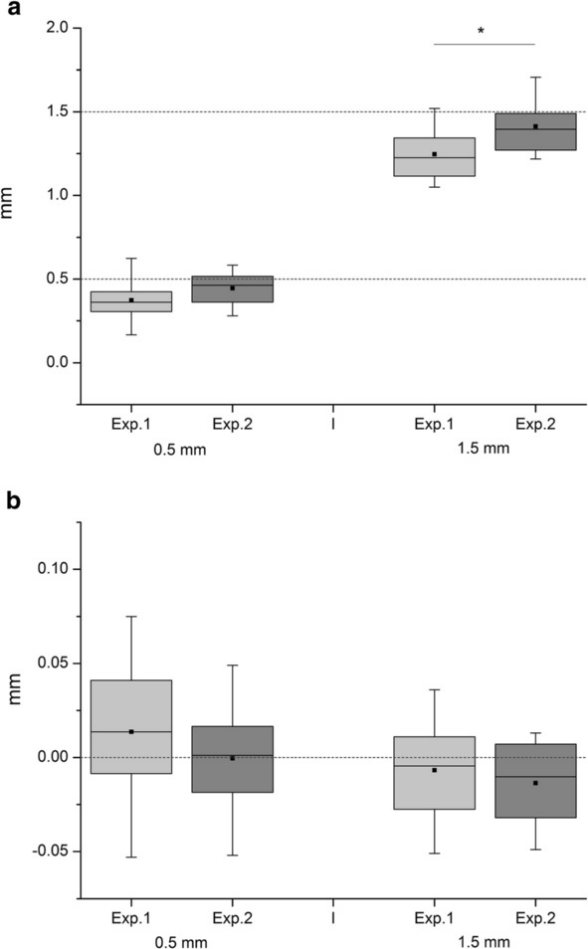
Analysis of the achieved depths. **a** The depths achieved by the two experimenters stay under the preset limits of 0.5 mm and 1.5 mm respectively. The less experienced person (Exp. 1) created a slightly but significantly lower depth in comparison to the expert (Exp. 2) when 1.5 mm depth was intended. Thus, the surgical instrument qualifies as secure in use, irrespective of the expertise of the user. **b** The levels of the inner part of the defects were cleared by the front mill. The results show small deviations from the reference grooves created by the crown mill (Fig. 6a) for both experimenters. Note the scale with steps of 0.01 mm in contrast to (**a**)

The depths of the created defects ranged from less than 0.5 and 1.5 mm for both operators, the less experienced and the expert, in the mean (Fig. 6a). The latter showed the lower deviations of 0.049 mm (set 0.5 mm) and 0.097 mm (set 1.5 mm) measured from the level of the circular groove made by the crown mill to the upper rim of the cartilage. In the case of the 1.5 mm defect, the result of the operators showed a difference of 0.163 mm (*p* = 0.018). The centres of the defects created by the front mill showed a low deviation from −0.013 mm (less) to 0.014 mm (deeper) (Fig. 6b).

**Fig. 7:**
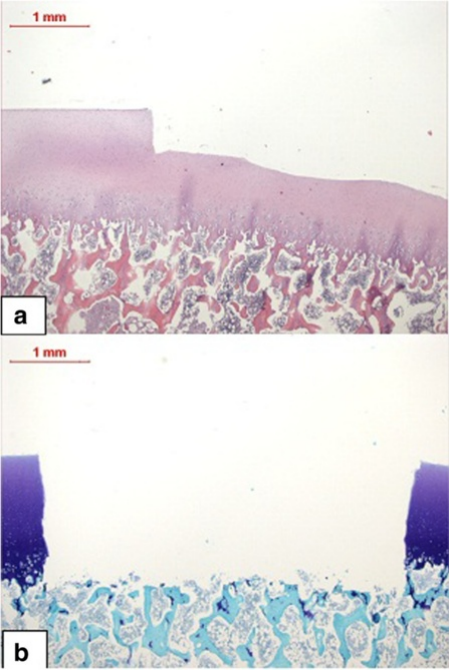
Histology of the cross sections. The cross sections of a 0.5-mm (**a**) and 1.5-mm (**b**) defect after HE and toluidine blue staining, respectively, show precise and smooth sides of the cartilage walls. The bottoms seem plane despite the slight undulating surface of the cartilage (**a**) and irregularities of the uncovered cancellous bone (**b**). The vertical sides and the bottom are perpendicular at the corners. Magnification 2.5×

The shapes of the defects show a perpendicular junction of the plain at the bottom and the sides of the cylindrical groove (Fig.7).

### Validation of the harvesting tool

The plugs were isolated after 7.42 ± 2.28 min in the mean.

The sides showed a mean deviation of −0.63° (CI: −1.33°; 0.08°) in 80 cases with a value of 0° for parallelism; thus, the punch and the whole apparatus worked consistently for all examined specimens. A critical geometry is the relation of the slope of the articular surface to the axis of the cylindrically shaped plug, which should be 90° in the best case. Deviations were calculated with 4.86° (CI: 4.154°; 5.573°) from the rectangularity (Fig. 5c).

Sterility was given in all cases (*n* = 80).

## Discussion

Reproducibility and standardisation of procedures in experimental surgery has more limitations the smaller the animals are, especially when articular cartilage is examined. But experiments with large animals are expensive in comparison to smaller ones like rodents or rabbits. A harmonisation of surgical procedures could deliver comparability between various experiments published in literature, leading to fewer animals needed in the end. Mainly regulatory requirements can be met if tools for standardisation are provided for surgeons, experimenters and lab personnel. But the quality of the particular scientific study will also benefit from tools that enable secure and quick work with animals and samples, respectively.

Information on tools used for creating defects in cartilage layer is given in publications of studies in the field [14, 15], and some authors describe the surgical technique in particular for validation [19, 20]. Frequently, the curettage is the recommended tool for creating the defects in combination with knives or circular cutting devices to determine the edges of the defect [19, 20]. The curette seems to be appropriate for removing tissue of the inner part of the defect. But its use is limited on the edges as its diameter has to be smaller than the diameter of an intended circular defect leading to irregular edges of the defect when looked at from above [20]. Seen from the side, the required perpendicularity between the sides and the bottom of the defect is not always possible. This is also the case if the curette has an offset (15°) [20] and bits of tissue may be found at the bottom depending on the technique used [19, 20]. If tissue remains in the “corners”, thus creating an inclined plane, it is a possibility that the implant slips out through the shear forces arising from the joint while moving. Bits of tissue are also found in the inner part of the defects after the use of a curette if e.g. the applied force was not strong enough [20]. However, the correct geometry of the defect is crucial for the stability of the construct. It is either glued, sutured or covered with a periosteal flap [26, 15], because the construct has to be protected through stable flanks for shouldering [18], and sutures need to find anchorage. Thus, primary stability of the implant can be supported by an appropriate quality of the defect avoiding a dislocation of the implanted construct. Punches or trephines are also used to create the defects as described [14, 15] to deliver well-shaped defects with regard to the defect edges. But controlling the depth is not always possible in situ during surgery due to the parallax of view in deeper levels. The removal of the core of the defect cylinder can be difficult if the bottom of the defect core stays in contact with the substrate. Chondral defects are prepared by custom-made instruments like an angulated raspatorium [14]. However, circular punches or trephines provide no centring components like drilling tools and depth control in high resolution. Both specifications are implemented in the presented instrument: a precise and reproducible centring and adjustment of the depth in 0.05-mm steps.

**Fig. 8:**
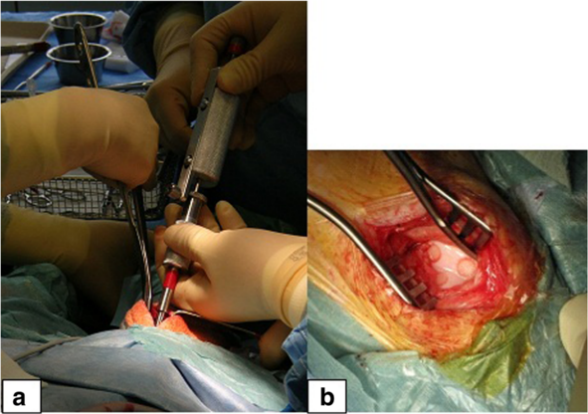
Use of the instruments in vivo. **a** The crown and front mill were applied on more than 60 Göttingen Minipigs. The trochlea is exposed clearly intraoperatively when the animal is lying on its back with a direct approach through the split patellar ligament. **b** Four chondral defects (one not visible) are created in the facets of the trochlea of a right knee joint of a Göttingen Minipig 6 mm in diameter. The defects are prepared, ready for the implantation of constructs later on. Sharp edges and rectangularity at the bottom of the defects will support the primary stability of the implants. The used instruments allow easy access due to their slim design, and the preparation of one defect is achieved in a few minutes

The developed and presented instruments fulfil the requirements of perpendicularity of the defect seen from the side, creation of straight and smooth surfaces of the sides and complete resection of the cartilage layer (Fig. 7). The instruments were used to create defects in the facets of the trochlea, offering a quite flat plane in the stifle joint (Fig. 8). Therefore, the convexity plays a lower role looking at the shape of the defect. Looking at the curvatures of e.g. condyles of a stifle joint, the shape changes due to different radii of the convexity of the condyles. We considered this situation in the construction of a concave front mill in another version (not presented here), but the problem remains unsolved, as one has to respect at least two different radii of the condyles in the sagittal plane and in the horizontal plane, respectively. However, the incongruence depends on the extension of a circular defect in terms of the relation between the diameter of the defect and the radius of the surface. In this case, the inner part of the defect can be taken out with the curette after the crown mill is used. As the width of the groove is only 1 mm, the form of the surface has only little influence on the shape.

The crucial issue in creating the defect is the depth. From the biological point of view, the anatomical edges are important like subchondral lamella or the tidemark. Both are hard to detect in the situs of operation, and surrogate criteria are used like the sound (“metal on stone” [20]) or training the surgeon to handle the curette properly [20]. However, experienced surgeons are in demand to create the defects. But the results show that the intended depths are not always reached depending on the applied technique [19, 20].

The presented instruments work independently of the force applied [20] as they are adjusted via positioning before use. Thus, reproducibility is given for experts and less experienced users as the data show in the validation experiment (Fig. 6). Hence, the disadvantage of this procedure may be seen in the fact that the depth of the defect has to be determined before creating it. But information on the height of the articular cartilage can be achieved by imaging or species-specific data [36, 37]. In addition, one can proceed stepwise respecting the expected depth as the cutting behaviour will change depending on the tissue while using the crown mill; also, isolated bleeding indicates a partial opening of the bone marrow. Thus, the advantage is that the crown mill is determining the final depth and can also be used as pilot instrument. The front mill will finish the defect while respecting the previously identified depth.

However, we have not compared our results to the results achieved through conventional techniques, neither before nor after healing processes. But we assume that the new technique we developed is less prone to influences such as the expertise of the surgeon executing the operation. We also believe that the new technique is likely to be a lot faster. Another disadvantage of the new technique could be that only defects of one predetermined diameter can be created during any one operation. Changing the diameter is thus not possible while operating. As the instruments were designed for the use in preclinical studies, where the size of the defect is determined before the operation, this is not a disadvantage. As our previous tests have shown, so far the diameter cannot be smaller than 4 mm. However, it is possible to create a crown mill and corresponding front mill of a larger diameter than the 6 mm we used.

Histopathological examination is indicated as the predominant outcome measurement tool in OA [26, 38, 39, 25, 16] and for regenerative therapeutic procedures, analysing the cartilage layer or regenerative tissue, the subchondral plate and the combination of both with other aspects [25]. Thus, the suitable harvesting of osteochondral samples is crucial for the preparation of histological sections. Proper sampling protocols need to respect comparable anatomical locations, or one has to focus on the most severe lesions [40, 25, 41, 42]. There are up to 12 regions requested for examination in a medial tibial condyle if a complex analysis is required, including several issues like biochemistry, biomechanics, gene expression, etc. [25]. The presented tool for the harvesting of osteochondral samples allows the isolation of several specimens even if they lie close together (Fig. 5). While sawing or grinding creates debris in the process, punching separates the tissue without leaving any debris. More importantly, it seems that the fact that the edges of the samples are smooth and defined, frayed and destroyed texture and cells can be avoided. Thermal alterations of the tissue can also be excluded as punching is executed under the application of PBS which, in addition, avoids drying out of the sample.

The presented punching procedure allows the exact harvesting of regenerated tissue or tissue originating from other locations, as the used apparatus allows the individual adjustment of the punch as desired. In combination with a 6-mm defect in diameter, a 5-mm sample in diameter can be extracted exactly as shown (Fig. 5). Further preparations of the articular layer on top are possible, as contamination is excluded by the used of “no touch” technique. Thus, a good starting point is also given for gene expression analysis or cell culture. The latter is possible as sterile preparation can be guaranteed.

In conclusion, we can provide new instruments and procedures for standardisation of creating defects in vitro and in vivo and for exact harvesting of samples in a quick, secure and sterile manner.

## Footnotes

1 Collagen I scaffold without cells, provided by Amedrix, Esslingen, Germany.

## References

[1] ASTM F2451-05(2010), Standard Guide for *in vivo* Assessment of Implantable Devices Intended to Repair or Regenerate Articular Cartilage. ASTM International, West Conshohocken, PA, 2010, http://www.astm.org; access July 13th 2015.

[2] EMEA/CHMP/410869/2006, Guideline on human cell-based medicinal products. European Medicines Agency, Canary Wharf, London, UK, 2008, http://www.emea.europa.eu; access July 13th 2015.

[3] Abedian R, Willbold E, Becher C, Hurschler C (2013). In vitro electro-mechanical characterization of human knee articular cartilage of different degeneration levels: a comparison with ICRS and Mankin scores. J Biomech.

[4] Fohr P, Hautmann V, Prodinger P, Pohlig F, Kaddick C, Burgkart R (2012). Design of a high-dynamic closed-loop controlled cartilage test system. Orthopade.

[5] Katta J, Stapleton T, Ingham E, Jin ZM, Fisher J (2008). The effect of glycosaminoglycan depletion on the friction and deformation of articular cartilage. Proc Inst Mech Eng H.

[6] Stoffel M, Yi JH, Weichert D, Zhou B, Nebelung S, Muller-Rath R (2012). Bioreactor cultivation and remodelling simulation for cartilage replacement material. Med Eng Phys.

[7] Bell CJ, Ingham E, Fisher J (2006). Influence of hyaluronic acid on the time-dependent friction response of articular cartilage under different conditions. Proc Inst Mech Eng H.

[8] Forster H, Fisher J (1996). The influence of loading time and lubricant on the friction of articular cartilage. Proc Inst Mech Eng H.

[9] Gleghorn JP, Doty SB, Warren RF, Wright TM, Maher SA, Bonassar LJ (2010). Analysis of frictional behavior and changes in morphology resulting from cartilage articulation with porous polyurethane foams. J Orthop Res.

[10] Katta J, Jin Z, Ingham E, Fisher J (2009). Effect of nominal stress on the long term friction, deformation and wear of native and glycosaminoglycan deficient articular cartilage. Osteoarthritis Cartilage.

[11] Schwarz ML, Schneider-Wald B, Krase A, Richter W, Reisig G, Kreinest M (2012). Tribological assessment of articular cartilage. A system for the analysis of the friction coefficient of cartilage, regenerates and tissue engineering constructs; initial results. Orthopade.

[12] Wimmer MA, Grad S, Kaup T, Hanni M, Schneider E, Gogolewski S (2004). Tribology approach to the engineering and study of articular cartilage. Tissue Eng.

[13] Chu CR, Szczodry M, Bruno S (2010). Animal models for cartilage regeneration and repair. Tissue Eng Part B Rev.

[14] Gotterbarm T, Breusch SJ, Schneider U, Jung M (2008). The minipig model for experimental chondral and osteochondral defect repair in tissue engineering: retrospective analysis of 180 defects. Lab Anim.

[15] Jubel A, Andermahr J, Schiffer G, Fischer J, Rehm KE, Stoddart MJ (2008). Transplantation of de novo scaffold-free cartilage implants into sheep knee chondral defects. Am J Sports Med.

[16] Schneider-Wald B, von Thaden AK, Schwarz ML (2013). Defect models for the regeneration of articular cartilage in large animals. Orthopade.

[17] Behrens P, Bosch U, Bruns J, Erggelet C, Esenwein SA, Gaissmaier C (2004). Indications and implementation of recommendations of the working group “Tissue Regeneration and Tissue Substitutes” for autologous chondrocyte transplantation (ACT). Z Orthop Ihre Grenzgeb.

[18] Steinwachs MR, Erggelet C, Lahm A, Guhlke-Steinwachs U (1999). Clinical and cell biology aspects of autologous chondrocytes transplantation. Unfallchirurg.

[19] Drobnic M, Radosavljevic D, Cor A, Brittberg M, Strazar K (2010). Debridement of cartilage lesions before autologous chondrocyte implantation by open or transarthroscopic techniques: a comparative study using post-mortem materials. J Bone Joint Surg Br.

[20] Mika J, Clanton TO, Pretzel D, Schneider G, Ambrose CG, Kinne RW (2011). Surgical preparation for articular cartilage regeneration without penetration of the subchondral bone plate: in vitro and in vivo studies in humans and sheep. Am J Sports Med.

[21] Redman SN, Oldfield SF, Archer CW (2005). Current strategies for articular cartilage repair. Eur Cell Mater..

[22] Richter W, Diederichs S (2009). Regenerative medicine in orthopaedics. Cell therapy—tissue engineering—in situ regeneration. Orthopade.

[23] Gudas R, Kalesinskas RJ, Kimtys V, Stankevicius E, Toliusis V, Bernotavicius G (2005). A prospective randomized clinical study of mosaic osteochondral autologous transplantation versus microfracture for the treatment of osteochondral defects in the knee joint in young athletes. Arthroscopy.

[24] Schneider U, Schmidt-Rohlfing B, Gavenis K, Maus U, Mueller-Rath R, Andereya S (2011). A comparative study of 3 different cartilage repair techniques. Knee Surg Sports Traumatol Arthrosc.

[25] Little CB, Smith MM, Cake MA, Read RA, Murphy MJ, Barry FP (2010). The OARSI histopathology initiative—recommendations for histological assessments of osteoarthritis in sheep and goats. Osteoarthritis Cartilage..

[26] Brehm W, Aklin B, Yamashita T, Rieser F, Trub T, Jakob RP (2006). Repair of superficial osteochondral defects with an autologous scaffold-free cartilage construct in a caprine model: implantation method and short-term results. Osteoarthritis Cartilage.

[27] Gavenis K, Schneider U, Maus U, Mumme T, Muller-Rath R, Schmidt-Rohlfing B (2012). Cell-free repair of small cartilage defects in the Goettinger minipig: which defect size is possible?. Knee Surg Sports Traumatol Arthrosc.

[28] Gille J, Kunow J, Boisch L, Behrerns P, Bos I, Hoffmann C, Köller W, Kurz B (2010). Cell-laden and cell-free matrix-induced chondrogenesis versus microfracture for the treatment of articular defects: a histological and biomechanical study in sheep. Cartilage.

[29] Goodrich LR, Hidaka C, Robbins PD, Evans CH, Nixon AJ (2007). Genetic modification of chondrocytes with insulin-like growth factor-1 enhances cartilage healing in an equine model. J Bone Joint Surg Br.

[30] Shortkroff S, Barone L, Hsu HP, Wrenn C, Gagne T, Chi T (1996). Healing of chondral and osteochondral defects in a canine model: the role of cultured chondrocytes in regeneration of articular cartilage. Biomaterials.

[31] Lahm A, Kreuz PC, Oberst M, Haberstroh J, Uhl M, Maier D (2006). Subchondral and trabecular bone remodeling in canine experimental osteoarthritis. Arch Orthop Trauma Surg.

[32] Pastoureau P, Leduc S, Chomel A, De Ceuninck F (2003). Quantitative assessment of articular cartilage and subchondral bone histology in the meniscectomized guinea pig model of osteoarthritis. Osteoarthritis Cartilage.

[33] Orth P, Cucchiarini M, Kohn D, Madry H (2013). Alterations of the subchondral bone in osteochondral repair—translational data and clinical evidence. Eur Cell Mater..

[34] Pickard JE, Fisher J, Ingham E, Egan J (1998). Investigation into the effects of proteins and lipids on the frictional properties of articular cartilage. Biomaterials.

[35] Podbielski AH, Herrmann MH, Kniehl EH, Mauch HH, Rüssmann HH, (DGHM) DGfHuM (2007). MiQ: Qualitätsstandards in der mikrobiologisch-infektiologischen Diagnostik.

[36] Frisbie DD, Cross MW, McIlwraith CW (2006). A comparative study of articular cartilage thickness in the stifle of animal species used in human pre-clinical studies compared to articular cartilage thickness in the human knee. Vet Comp Orthop Traumatol.

[37] Koff MF, le Chong R, Virtue P, Chen D, Wang X, Wright T (2010). Validation of cartilage thickness calculations using indentation analysis. J Biomech Eng.

[38] Custers RJ, Saris DB, Dhert WJ, Verbout AJ, van Rijen MH, Mastbergen SC (2009). Articular cartilage degeneration following the treatment of focal cartilage defects with ceramic metal implants and compared with microfracture. J Bone Joint Surg Am.

[39] Jung M, Kaszap B, Redohl A, Steck E, Breusch S, Richter W (2009). Enhanced early tissue regeneration after matrix-assisted autologous mesenchymal stem cell transplantation in full thickness chondral defects in a minipig model. Cell Transplant.

[40] Appleyard RC, Burkhardt D, Ghosh P, Read R, Cake M, Swain MV (2003). Topographical analysis of the structural, biochemical and dynamic biomechanical properties of cartilage in an ovine model of osteoarthritis. Osteoarthritis Cartilage.

[41] Young AA, Appleyard RC, Smith MM, Melrose J, Little CB (2007). Dynamic biomechanics correlate with histopathology in human tibial cartilage: a preliminary study. Clin Orthop Relat Res..

[42] Young AA, McLennan S, Smith MM, Smith SM, Cake MA, Read RA (2006). Proteoglycan 4 downregulation in a sheep meniscectomy model of early osteoarthritis. Arthritis Res Ther.

